# Predicted PK/PD inadequacy of amoxicillin delivered in drinking water against respiratory pathogens in post-weaning piglets: can time-restricted dosing improve animals’ exposure and potential treatment efficacy?

**DOI:** 10.1016/j.vas.2026.100698

**Published:** 2026-05-14

**Authors:** Marine Lacampagne, Gwendoline Hervé, Lucie Claustre, Natasha Jourdannaud, Marlène Lacroix, Béatrice Roques

**Affiliations:** a INTHERES, Université de Toulouse, INRAE, ENVT, 23 chemin des Capelles, Toulouse, France; b IFIP, Institut du Porc, 9 boulevard du Trieux 35740 Pacé, France

**Keywords:** Amoxicillin, Pharmacokinetics, Modeling, Piglets, Drinking-water, MIC, Efficacy

## Abstract

Collective treatments via drinking water allow rapid and targeted treatment of many animals as soon as disease is detected, but inter-animal variability in drinking behaviour can lead to over- or under-exposure. We therefore developed a pharmaco-statistical model integrating real-time individual water consumption to simulate amoxicillin plasma concentrations and assess actual treatment efficacy.

One hundred fifty piglets received amoxicillin (10 mg/kg/day, 2.5–3 days) via drinking water under continuous, daytime, or night-time treatment, and water and blood samples were collected. Using individual drinking profiles and pharmacokinetic parameters, we simulated plasma concentrations in a virtual population of 5000 piglets per modality to compared with the obtained actual plasma concentrations. We then estimated the proportion of adequately treated animals by comparing modelled plasma concentrations with MICs for five major respiratory pathogens.

Amoxicillin treatment was effective in at least 80% of piglets only for one of the five targeted pathogens, *Streptococcus suis*. These findings suggest that it may be necessary to evaluate other antibiotics using the same modelling approach to improve treatment of *Glaesserella parasuis, Actinobacillus pleuropneumoniae, Pasteurella multocida*, and *Bordetella bronchiseptica*. This strategy could also be extended to other species treated collectively via drinking water, such as poultry.

## Highlights

• Model integrates individual drinking profiles to simulate amoxicillin exposure.
• Inter-animal drinking-behavior variability leads to inconsistent plasma levels.
• Time-restricted dosing (day/night) does not improve group-level efficacy.
• Adequate exposure achieved only against *Streptococcus suis.*
• Approach adaptable to other antibiotics and species treated via drinking water.

## Graphical abstract

**Figure:**
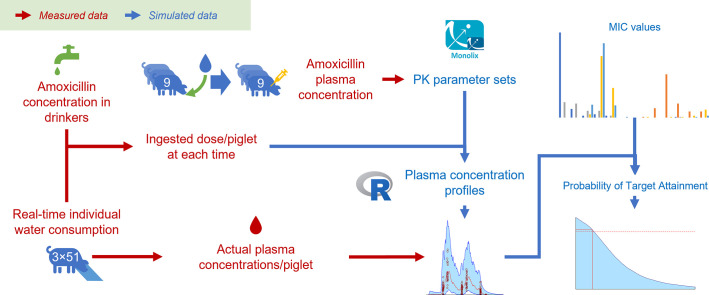

## 1 Background

Reducing antibiotic use in veterinary medicine is one of the key strategies to address antibiotic resistance, and European initiatives have led to substantial declines in usage, particularly in pig farming. In France, for instance, the pork industry reduced antibiotic consumption by more than 70% between 2010 and 2022, with a particularly marked 92% decrease in post‑weaning piglets (Poissonnet et al., 2025). As a result, treatment levels are now similar across suckling piglets, post‑weaning piglets and sows (Poissonnet et al., 2025). Before this recent decline, the post-weaning stage had long been the most antibiotic-consuming phase in France (Poissonnet et al., 2022), as well as in several other European countries (Sjölund et al., 2016). Despite this progress, the post‑weaning period remains a critical phase for disease expression, and recent national data show that respiratory disease has become the leading reason for antibiotic use at this stage, accounting for 43% of treatments in 2022 (Poissonnet et al., 2025). When treatment is required, amoxicillin remains one of the main antibiotics used to manage systemic infections such as *Streptococcus suis*, as well as respiratory infections caused by *Actinobacillus pleuropneumoniae* or *Pasteurella multocida* (Burch & Sperling, 2018).

To ensure the effectiveness of these treatments, it has been shown that early/metaphylactic antibiotic treatments of sick animals but also of contaminated ones are the most effective (Lhermie et al., 2016; Vasseur et al., 2017). Given the high stocking density typical of pig farms, this strategy requires treating dozens or even hundreds of animals simultaneously over several days, which precludes individual administration by intramuscular or subcutaneous routes.

Collective treatments delivered orally via feed or drinking water make it possible to medicate large numbers of animals as soon as disease is detected in a few. However, in the context of prudent antimicrobial use of antimicrobials in veterinary medicine, the oral administration of antibiotics to groups of animals via feed or drinking water is a major concern in Europe, and oral group medications were classified as high risk for selecting resistance, the risk being greater for feed than for water (European Medicines Agency (EMA), 2019). Medicated feed requires specific manufacturing processes that can cause delays, inadequate batch sizes, or storage issues, and these in turn may lead to contamination of non-medicated feed (Filippitzi et al., 2016). In addition, the altered behaviour of sick animals can result in underexposure to antibiotics administered in feed, whereas the impact on water intake appears to be smaller (Gagnon et al., 2018; Little et al., 2019; Thomas et al., 2021).

By contrast, antibiotic delivery via drinking water offers greater flexibility because medicated water can be prepared on site with adjustable doses and volumes. Nevertheless, plasma antibiotic concentrations can vary considerably across animals during collective treatments. Although this variability seems less pronounced with drinking-water treatments than with medicated feed (Soraci et al., 2014), it may still cause under-dosing, with reduced clinical efficacy and increased resistance selection, or over-dosing, which raises the risks of toxicity and residue persistence at slaughter.

Soraci et al. (2014) and Little et al. (2019) identified three main sources of interindividual variability in exposure during collective antibiotic treatments via drinking water: variability in the dose actually administered, interindividual variability in antibiotic pharmacokinetics (PK), and interindividual variability in water consumption.

Human error, such as miscalculations during preparation, or technical faults in dosing pumps due to poor maintenance, can contribute substantially to under- or over-dosing (Georgaki et al., 2023). The antibiotic’s physicochemical properties (solubility, stability, homogeneity in water) as well as interactions with water disinfection processes can also alter the concentration effectively delivered (Little et al., 2019; Vandael et al., 2019).

Interindividual PK variability further affects antibiotic exposure because physiological differences influence bioavailability, absorption, metabolism, and excretion (Bousquet-Melou et al., 2017). Food intake and the presence of infectious diseases may also modify bioavailability (Godoy et al., 2010; Jensen et al., 2006; Nielsen & Gyrd-Hansen, 1996). However, when antibiotics are administered in drinking water, PK variability tends to contribute less to differences in plasma concentrations than variability in dose intake, which remains the dominant factor influencing exposure (Chassan et al., 2021; Ferran et al., 2020).

Dose intake is directly linked to drinking behaviour, which is the primary source of variability in collective water treatments. In pigs, water consumption follows a circadian rhythm synchronised with the light–dark cycle, with higher consumption during daylight. Yet profiles vary widely due to numerous factors. Pigs tend to drink mainly around feeding times, with roughly 75% of daily intake occurring then, including about 25% before meals (Bigelow & Houpt, 1988). Even within a single cohort, drinking patterns may differ between pens (Little et al., 2023). Profiles may be unimodal or bimodal, most often showing a dominant afternoon peak and, in some cases, a secondary morning peak that becomes more pronounced as pigs grow older and heavier (Larsen & Pedersen, 2022; Little et al., 2022).

At the group level, water intake increases with age and weight (Gagnon et al., 2018; Little et al., 2022). Social hierarchy can shape individual intake patterns as well, with subordinate animals having more limited access to drinkers than dominant ones, resulting in uneven antibiotic exposure (Soraci et al., 2014).

Environmental conditions also play a role. High ambient temperatures increase water intake, and part of this may be due to spillage used as a thermoregulatory behaviour under heat stress (Gagnon et al., 2018; Ramonet et al., 2019). Spillage can be exacerbated when inappropriate bowl drinkers are used (Madsen et al., 2005) or when drinker flow rates are not properly adjusted (Massabie et al., 2014).

In sum, although collective treatments via drinking water are widely used in pig farming because of their flexibility, multiple sources of variability influence antibiotic exposure, making optimal use at the group level difficult to achieve.

Several modelling approaches have already been developed to characterise the variability in antibiotic exposure during drinking‑water medication. In poultry, Temmerman et al. (2021) proposed a drinking‑behaviour pharmacokinetic model that accounts for day–night variation in water intake, although this approach relied on simplified intake assumptions and a limited number of animals. In pigs, more advanced modelling work has used RFID‑derived drinking‑behaviour profiles to simulate exposure under different dosing strategies (Chassan et al., 2021; Hernández et al., 2024), but these studies were based on fully simulated concentrations and often explored dosing modalities that remain difficult to implement under actual farm conditions. A field‑based approach was explored in lambs by Ferran et al. (2020), who monitored both individual drinking behaviour and plasma concentrations to characterise inter‑animal variability.

Building on these complementary efforts, the present study aims to bridge the gap between theoretical modelling and farm reality by integrating real‑time individual water‑intake signals from 153 post‑weaning piglets into a PK model and comparing its predictions with their measured amoxicillin plasma concentrations. This enables to build a pharmaco‑statistical model directly comparable with field plasma concentrations. The model was then used to assess PK/PD target attainment for five major respiratory pathogens under three practically applicable dosing modalities (continuous, daytime‑restricted and night‑time‑restricted), providing a robust and operational basis for optimising collective treatments in post‑weaning piglets.

## 2 Materials and methods

### 2.1 Animals

All experiments were conducted on post-weaning piglets with *ad libitum* access to water and feed. Piglets were supplied by GAEC de Calvignac (Danbred–Duroc crossbred, St Vincent d’Autejac, France) for the laboratory experiment and by IFIP (Landrace × Large White × Piétrain, French Pork and Pig Institute, Romillé, France) for the farm experiment. The protocols were authorised by the French Ministry of Research under the number #44369_202308070921823 for the laboratory experiment carried out at the INTHERES animal facility, and #46144_2023112916569025 for the experiment performed at the IFIP experimental farm.

### 2.2 PK of amoxicillin

The study was performed on nine post-weaning piglets (four males and five females) aged 4 weeks on arrival at the INTHERES facility. The animals were housed in collective pens and underwent a 1.5-week acclimatisation period before the experiment began. Throughout the study, including during drug administration and blood sampling, the piglets had *ad libitum* access to water and feed, with no fasting period prior to treatments.

The PK study followed a 3 × 3 crossover design. In this publication, we report only the leg concerning administration of amoxicillin (Cofamox 10®; Dopharma, Vair-sur-Loire, France) at 10 mg/kg body weight (BW). Amoxicillin was given as an oral bolus via spontaneous intake using a syringe. The mean (± SD) BW of the three piglets treated each week was 8.5 ± 1.8 kg, 12.9 ± 0.4 kg, and 23.5 ± 0.9 kg for weeks 1 to 3, respectively. Blood samples were collected before administration and at 15 min, 30 min, 1 h, 2 h, 3 h, 5.5 h, 7.5 h, 9.5 h, 12 h, and 24 h after oral administration.

In the fourth week, all piglets received an intravenous bolus of amoxicillin (Panpharma®; Luitré, France) at 10 mg/kg BW. Administration was performed via a catheter placed in the ear vein under anaesthesia [medetomidine (0.1 mL/kg, Medetor^ND^; Virbac, France) / tiletamine-zolazepam (0.05 mL/kg, Zoletil^ND^; Virbac, France)]. This intravenous administration served as a reference to determine the oral bioavailability of amoxicillin. Mean (± SD) BW at this stage was 27.7 ± 4.0 kg. Blood samples were collected before administration and 2 min, 5 min, 15 min, 45 min, 1.5 h, 3 h, 5 h, 8 h, and 10 h post-administration.

Each blood sample was collected in heparinised tubes, centrifuged at 3000 x *g* for 10 min at +4°C, and the recovered plasma were stored at -20°C until analysis.

### 2.3 Amoxicillin plasma concentrations and individual water consumption in experimental farm

Three groups of 51 post-weaning piglets (13.4 ± 1.6 kg BW, measured 4 days before the start of the experiment; i.e., at 7 weeks old), housed in three adjoining pens per group, were treated with Amoxipro 10% (Ceva Santé Animale, Libourne, France). The aim was to compare plasma amoxicillin concentrations under three treatment regimens.

The goal was, in a first half-room of 51 pigs, to set up a control ‘continuous treatment’ (from 5 pm to 5 pm) with a group target of 10 mg/kg BW/24 h for 3 days. The medicated water was renewed twice daily (8 am and 5 pm) to limit amoxicillin degradation.

The other two groups, housed in a separate room divided into two half-rooms sharing a common pump, received either a ‘night-time’ treatment (5 pm to 8 am) or a ‘daytime’ treatment (8 am to 5 pm) for 3 consecutive days. The aim for these two groups was to deliver the same total daily dose as the continuous treatment but concentrated within a restricted period, with untreated water provided outside treatment hours.

The metering pump was set to 5% for all groups. This setting means that the pump injected a volume of medicated stock solution corresponding to 5% of the total water flow entering the drinking‑water line. Piglets in each group were allocated to 3 pens of 17 piglets, each pen equipped with a single drinking trough. The flow rate of each trough was set to 1 L/min and checked when the animals entered the post-weaning stage. Individual water consumption was recorded in real time for all piglets in all pens from the day prior treatment until the third day after treatment, using water meters connected to the troughs and linked to radiofrequency identification chips in the piglets’ ear tags. Access to the drinking troughs allowed the visit of only one piglet at a time.

Blood samples were collected to monitor amoxicillin plasma concentrations. In each group, nine piglets per pen were sampled every morning for 3 days (continuous and night-time treatments) or 4 days (daytime treatment) after treatment began. The eight remaining piglets per pen were sampled each afternoon for 3 days across all groups. Blood samples were collected in heparinised tubes, centrifuged at 3000 × *g* for 10 min at +4°C, and plasma were stored at -20°C until analysis.

To monitor the actual administered dose, water samples were collected from each trough at several points during the treatment period: immediately after treatment began, in the morning before and after preparation of medicated water, and mid and late afternoon, before or after water renewal. Additional samples were taken from the dosing pump at the same times until treatment ended, to check for any degradation of amoxicillin between pump and troughs. Samples were also taken from the troughs once treatments had stopped. All water samples were stored at -20°C until analysis to verify the actual concentrations of amoxicillin administered and refine the PK analyses.

### 2.4 Amoxicillin assays

Amoxicillin plasma concentrations were determined by liquid chromatography–mass spectrometry using an Acquity ultra-performance liquid chromatography (UPLC®) system coupled to a Xevo® triple quadrupole mass spectrometer (Waters, Milford, MA, USA). Plasma samples (100 µL) were spiked with 400 µL of MeOH and 10 µL of IS amoxicillin-d4 at 1 µg/mL in H_2_O, then centrifuged for 10 min at 20,000 × *g* at +4°C. The supernatant (300 µL) was evaporated under nitrogen at 45°C and reconstituted in 100 µL of ammonium acetate buffer (25 mM, pH 5). Analytes (10 µL) were injected onto a C18 column (Acquity BEH C18, 2.1 × 50 mm, 1.7 µm; Waters) with an H_2_O/0.1% HCOOH–AcN gradient elution [t(0 → 1.9 min): 5% AcN; t(1.9 → 2 min): 30% AcN; t(2 → 4 min): 5% AcN)]. Amoxicillin and amoxicillin-d4 were ionised by electrospray in positive mode (ESI+) and detected in multiple reaction monitoring mode (MRM) with the following transitions: amoxicillin m/z 366 > 114 (Ecoll 22 eV) and amoxicillin-d4 m/z 370 > 114 (Ecoll 18 eV). For both the laboratory PK experiment and the experimental farm study, the method was validated with a calibration curve ranging from 0.01 to 5 µg/mL using a quadratic model weighted by 1/X^2^ and three QC samples (0.025, 0.25, and 2.5 µg/mL). Accuracy ranged from 105% to 106%, and intra- and inter-day coefficients of variation were below 10%. Amoxicillin concentrations in drinking water were quantified using the same chromatographic conditions with UV detection at 270 nm. Drinking-water samples (100 µL) were diluted with 100 µL of ampicillin (IS) at 100 µg/mL in H_2_O. Amoxicillin concentrations were estimated with a calibration curve ranging from 0.5 to 250 µg/mL using a linear model weighted by 1/X^2^. Accuracy assessed with three QC samples (1.5, 15, and 150 µg/mL) ranged from 102% to 104%, and intra-day coefficient of variation precision was below 4%.

### 2.5 PK analysis

**Fig. 1:**
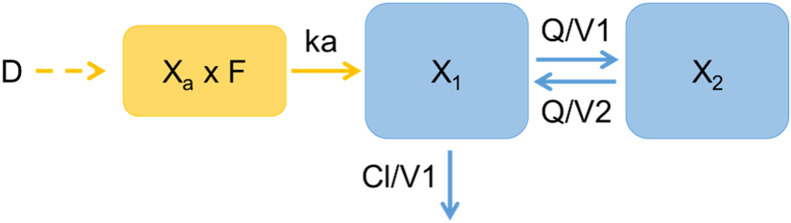
Bicompartmental model used to describe amoxicillin plasma concentrations. D: amoxicillin dose (mg/kg BW); X: quantity of amoxicillin (mg) in compartment ‘a’ for absorption, ‘1’ for the central compartment, and ‘2’ for the peripheral compartment; V_1_: volume of the central compartment; V_2_: volume of the peripheral compartment; Cl: elimination clearance; Q: inter-compartmental clearance; ka: rate constant of absorption; F: oral bioavailability.

For the laboratory experiment, compartmental analyses were carried out using a nonlinear mixed-effects model (NLME) with Monolix (Lixoft®, v.2023R1; Antony, France). The time course of amoxicillin concentrations after both intravenous and oral administration was fitted to a bicompartmental model with first-order absorption for the oral route (Fig. 1). Overall, this model structure was used to estimate population pharmacokinetic parameters and to simulate individual concentration-time profiles by incorporating the water consumption profiles recorded on farm. Values below the limit of quantification (LOQ) of 0.01 µg/mL were censored for the analysis. The model was described by the following parameters: V1, volume of the central compartment; V2, volume of the peripheral compartment; Cl, elimination clearance; Q, inter-compartmental clearance; ka, rate constant of absorption; and F, oral bioavailability.

Inter‑individual variability was estimated for all PK parameters. Several covariates (body weight, age, sex) were tested but did not improve model fit (−2LL, BIC). More complex structural models described in the amoxicillin literature (e.g., transit absorption models, additional distribution compartments) were also considered during model development. However, given the objective of generating parameter distributions for population‑level exposure simulations, and the predominance of drinking‑behaviour variability in this framework, a simpler bicompartmental model with first‑order absorption was retained for robustness and ease of reuse.

### 2.6 Modelling of plasma concentration profiles of amoxicillin administered via drinking water and evaluation of treatment efficacy

All modelling was performed using R version 4.4.2 (R Core Team, 2024, Vienna, Austria) within the RStudio® integrated development environment (RStudio Team, Boston, MA, USA, 2024).

Minimal inhibitory concentration (MIC) distribution frequencies for pig isolates of five major respiratory pathogens - *Pasteurella multocida, Actinobacillus pleuropneumoniae, Bordetella bronchiseptica, Glaesserella parasuis*, and *Streptococcus suis -* were retrieved from El Garch et al. (2016), based on the pan-European antibiotic susceptibility monitoring program VetPath.

By combining these MICs with data from the laboratory experiment (amoxicillin PK parameters) and the farm study (amoxicillin concentrations in water and plasma, and piglets’ water consumption), we developed a pharmaco-statistical model to determine whether the current treatment (i.e., continuous treatment) with amoxicillin via drinking water was sufficient to treat at least 80% of piglets against the five respiratory pathogens of interest, and whether an alternative dosing regimen could be proposed to achieve or improve this target, taking into account piglets’ drinking behaviour without altering the total daily dose administered.

#### 2.6.1 Randomised selection of PK parameters and modelling of antibiotic plasma concentrations

PK parameters from the laboratory experiment were used to generate a lognormal distribution for V1, V2, Cl, Q, and ka, and a logit-normal distribution for F. This allowed 100 sets of individual values for the six parameters to be sampled. Each drinking behaviour profile was then associated with these 100 sets, generating 51 piglets × 100 PK sets; i.e., 5100 plasma concentration profiles. Because drinking-behaviour traceability was lost for one pig in each batch (no detection by the sensors), we were ultimately able to simulate 5000 plasma concentration profiles.

To do this, the PK model selected in the previous step, namely a bicompartmental model with first-order absorption was used. The differential equations describing the amount of amoxicillin in the absorption compartment (X_a_) (Eq. (1)), the central compartment (X_1_) (Eq. (2)) and the peripheral compartment (X_2_) (Eq. (3)) were as follows (initial conditions were respectively D x F, 0 and 0, with D the Dose (see §2.6.2)):

(1) $$\frac{dX_{a}}{dt}=\;-ka\;\times X_{a}$$

(2) $$\frac{dX_{1}}{dt}=\;ka\;\times X_{a}-\left(\frac{Q}{V_{1}}-\frac{Cl}{V_{1}}\right)\times X_{1}+\frac{Q}{V_{2}}\times X_{2}$$

(3) $$\frac{dX_{2}}{dt}=\frac{Q}{V_{1}}\times X_{1}-\frac{Q}{V_{2}}\times X_{2}$$

These differential equations were resolved using the ode function of the *deSolve* R package, applying proportionality and superposition principles for each medicated-water intake.

#### 2.6.2 Theoretical and actual concentrations in drinking troughs and piglets’ drinking behaviour

To check that the model accurately predicted actual plasma concentrations, we calculated the actual dose of amoxicillin ingested by each piglet using concentrations measured in the troughs. The instantaneous ingested dose was calculated according to Eq. (4):

(4) $$\text{Dose}\;\left(\text{mg}/\text{kg}\right)=\frac{\text{quantity}\;\text{of}\;\text{water}\;\text{ingested}\;\left(\text{mL}\right)\;\times \;\text{actual}\;\text{antibiotic}\;\text{concentration}\;\left(\text{mg}/L\right)}{\text{bodyweight}\;\left(\text{kg}\right)}$$

Dose calculations used averaged trough amoxicillin concentrations alongside water-consumption and piglet-weight data. Each water intake was recorded via radiofrequency identification, enabling direct association between volume consumed and the piglet’s weight at that moment. Missing weight data were linearly interpolated (*na.approx*, R *zoo* package).

This initial step allowed us to compare, both visually and quantitatively, the distribution of the 5000 simulated plasma concentration profiles with the plasma concentrations actually measured in the 50 sampled pigs per batch. We found this comparison satisfactory and decided to continue using this PK model to simulate other dosing regimens.

We therefore carried out simulations using three administration modalities - continuous (8 am to 8 am), daytime (8 am to 5 pm) and night-time (5 pm to 8 am) - using a common target dose of 10 mg/kg/day for 5 days.

The antibiotic concentration required in water was calculated using Eq. (5):

(5) $$\text{Antibiotic}\;\text{concentration}\;\left(\text{mg}/L\right)=\frac{\text{targeted}\;\text{dose}\;\left(\text{mg}/\text{kg}/d\right)\times \;\text{portion}\;\text{reaching}\;\text{troughs}\;\left(\%\right)\;\times \;\text{mean}\;\text{piglet}s^{{}^{\prime }}\text{BW}\;\left(\text{kg}\right)}{\text{mean}\;\text{water}\;\text{consumption}\;\left(L/d\right)}$$

For both restricted treatment modalities, we assumed piglets consumed half of their daily water intake between 8 am and 5 pm, and the other half between 5 pm and 8 am.

Using the same water-consumption and body-weight data, and the same 100 PK parameter sets, we simulated 5000 new plasma concentration profiles per modality for a 5-day amoxicillin treatment. This enabled assessment of the efficacy of the three strategies via a pharmacokinetic/pharmacodynamic (PK/PD) indicator based on MIC.

#### 2.6.3 Randomized selection of bacteria MICs

MIC distribution frequencies reported by El Garch et al. (2016) for *Pasteurella multocida, Actinobacillus pleuropneumoniae, Bordetella bronchiseptica, Glaesserella parasuis*, and *Streptococcus suis* pig isolates were used as shown in Table 1.

**Table 1:** MIC distribution for *Pasteurella multocida, Actinobacillus pleuropneumoniae, Bordetella bronchiseptica, Glaesserella parasuis*, and *Streptococcus suis* pig isolates against amoxicillin.

**MIC (mg/L)**	2^−5^	2^−4^	2^−3^	2^−2^	2^−1^	2^0^	2^1^	2^2^	2^3^	2^4^	2^5^	2^6^	2^7^
***P. multocida*** **(n = 152)**	0	0	19	110	18	1	0	0	0	0	0	1	3*
***A. pleuropneumoniae*** **(n = 158)**	0	0	5	91	44	0	0	1	0	1	1	3	12*
***B. bronchiseptica*** **(n = 117)**	0	0	0	0	0	0	0	1	14	64	21	9	9*
***G. parasuis*** **(n = 68)**	23*	21	10	10	3	0	0	0	0	0	1	0	0*
***S. suis*** **(n = 151)**	126*	13	3	5	3	0	0	0	0	0	1	0	0*

Values marked with an asterisk (*) indicate MICs below the lowest concentration tested (left of the table) or above the highest concentration tested (right of the table).

These values were combined with the 5000 plasma concentration profiles obtained previously, enabling calculation of 5000 PK/PD indices for each bacterium. The selected PK/PD index was AUC_24h_/MIC because the efficacy of amoxicillin - a time-dependent antibiotic from the β-lactam family - is better described by this index than by T>MIC (Lees et al., 2015).

We then tested two scenarios based on data from Bousquet-Melou et al. (2017): a bacteriostatic effect, using an AUC_24h_/MIC threshold of 28 h, and a bactericidal effect, using a threshold of 45 h.

## 3 Results

### 3.1 PK parameters of amoxicillin

**Fig. 2:**
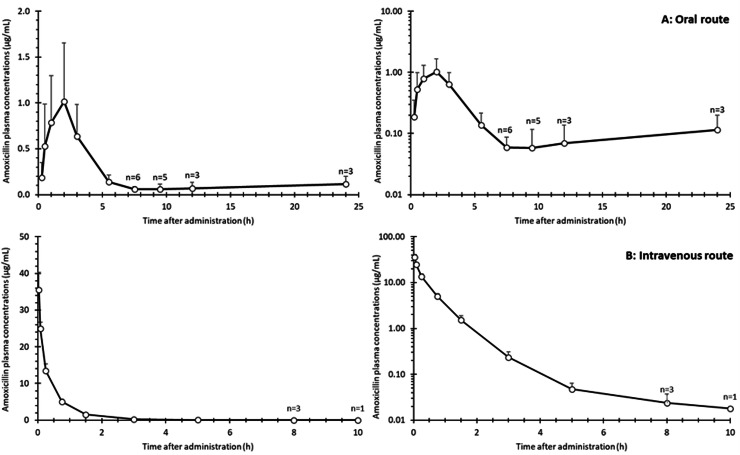
Mean (± SD) plasma concentrations of amoxicillin following a single oral administration (Cofamox 10®, 10 mg/kg BW, panel A) or intravenous administration (Amoxicillin Panpharma®, 10 mg/kg BW, panel B) in nine piglets. Left and right panels display the data on linear and logarithmic scales, respectively. ‘n = x’ indicates the number of piglets with plasma concentrations above the LOQ at each time point when n < 9.

PK profiles of amoxicillin were obtained for nine piglets following successive oral and intravenous administrations. The mean (± SD) time-course profiles of amoxicillin plasma concentrations are shown in Fig. 2. Overall, these profiles illustrated the low variability after IV dosing and the greater dispersion and lower peak concentrations observed after oral administration.

For all nine piglets, amoxicillin plasma concentrations remained above the LOQ of the assay (0.01 µg/mL) until 5.5 h after oral administration and 5 h after intravenous administration. The mean (CV (%)) population PK parameters are presented in Table 2.

**Table 2:** Mean (CV (%)) population PK parameters of amoxicillin derived from intravenous (Amoxicillin Panpharma®, 10 mg/kg BW) and oral (Cofamox 10®, 10 mg/kg BW) administrations in nine piglets.

PK parameters	Units	Estimates
Cl	L/h.kg	0.79 (6.9)
Q	L/h.kg	0.60 (11.58)
V1	L/kg	0.28 (3.47)
V2	L/kg	0.17 (5.47)
Ka	1/h	0.41 (29.36)
F	%	25 (47)

Cl: clearance of the central compartment; Q: inter-compartmental clearance; V1: volume of the central compartment; V2: volume of the peripheral compartment; ka: absorption rate constant; F: oral bioavailability.

### 3.2 Individual water consumption in experimental farm

On the first day of recording, piglets consumed an average of 1.6 L, 1.4 L and 1.3 L of water over 24 h in the continuous, daytime and night-time groups, respectively.

**Fig. 3:**
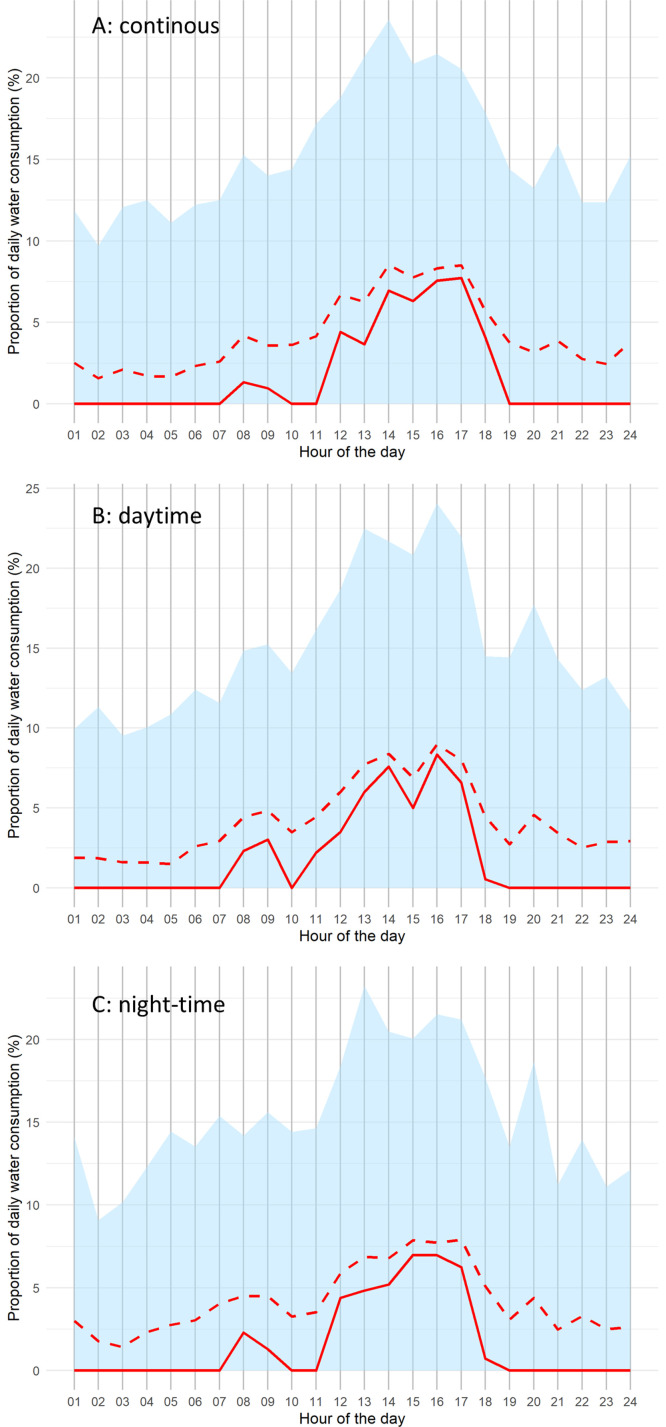
Hourly distribution of daily water consumption expressed as a proportion of total daily intake (%) over 8 days for piglets in the continuous (A), daytime (B) and night-time (C) modalities. The red solid line represents the median and the red dashed line represents the average water consumption over the entire period for all piglets for each hour of the day. The blue area represents the distribution of values between the 5^th^ and 95^th^ percentiles.

Fig. 3 shows the proportion of total daily water intake consumed each hour across the recorded period for each piglet in the three treatment modalities (continuous, daytime, and night-time). Across all modalities, piglets exhibited broadly similar daily drinking patterns, with a modest peak between 7 am and 10 pm. Most water consumption occurred between noon and 7 pm, representing 51.8% ± 13.6%, 50.5% ± 13.5%, and 48.1% ± 16.5% of daily intake for the continuous, daytime, and night-time modalities, respectively. Overall, the three modalities showed broadly similar circadian drinking patterns and comparable variability.

### 3.3 Amoxicillin concentration in drinking troughs

During the experiment, the amoxicillin solution in the metering pump appeared homogeneous, and no deposit was observed in the drinking troughs. Based on the measured concentrations of amoxicillin in both the metering pump and the troughs, we estimated that only 69% ± 20% of the theoretical dose actually reached the troughs (only the values in bold in Table 3 were included in this calculation). Detailed results for each pump (common pump for the daytime and night-time modalities) showing the percentage of the theoretical amoxicillin concentration found in the troughs during the different treatment periods are provided in Table 3. After treatment ended, amoxicillin rapidly disappeared from the watering system.

**Table 3:** Mean (± SD) percentage of the theoretical concentrations of amoxicillin found in drinking troughs during treatment of 153 piglets via drinking water (Amoxipro 10%, 10 mg/kg BW/d). Values in bold were used to estimate the proportion of the theoretical dose that reached the troughs.

Experiment stages	Sampling time	Mean (± SD) % [Min–Max] of the theoretical concentration	
		Continuous treatment	Daytime and night-time treatments
1^st^ day	Treatment beginning (5 pm)	22% ± 31% [2–57]	116% ± 23% [95–142]
2^nd^ day	8 am	-	**4% ± 3% [1–7]**
	10:30 am	**52% ± 26% [23–71]**	**55% ± 4% [51–60]**
	4 pm	**78% ± 3% [77–81]**	**46% ± 3% [42–49]**
	5 pm	**79% ± 5% [75–85]**	**36% ± 1% [35–37]**
3^rd^ day	8 am	-	**73% ± 3% [71–77]**
	9:30 am	**74% ± 5% [68–79]**	**107% ± 13% [93–119]**
	3:30 pm	**85% ± 4% [82–90]**	**76% ± 2% [74–78]**
	5 pm	**75% ± 10% [65–86]**	**77% ± 3% [74–79]**
4^th^ day	8 am	-	**82% ± 2% [80–83]**
	9:30 am	3% ± 1% [2–5] b	**32% ± 9% [24–42]**
	3:30 pm	1% ± 0% [0–1] b	**73% ± 2% [71–75]**
	5 pm	BLQ ab	**78% ± 1% [77–80]**
5^th^ day	9:30 am	BLQ ab	1% ± 2% [0–4] b

a BLQ = below the limit of quantification (0.5 µg/mL).

b After the end of treatment, it was not possible to calculate a percentage of theoretical concentration in the trough relative to the pump concentration because the theoretical concentration in the pump was zero. The percentages shown therefore represent the amount of amoxicillin remaining in the troughs relative to the concentration measured before treatment ended.

### 3.4 Model fitting with actual data obtained after administration via drinking water

**Fig. 4:**
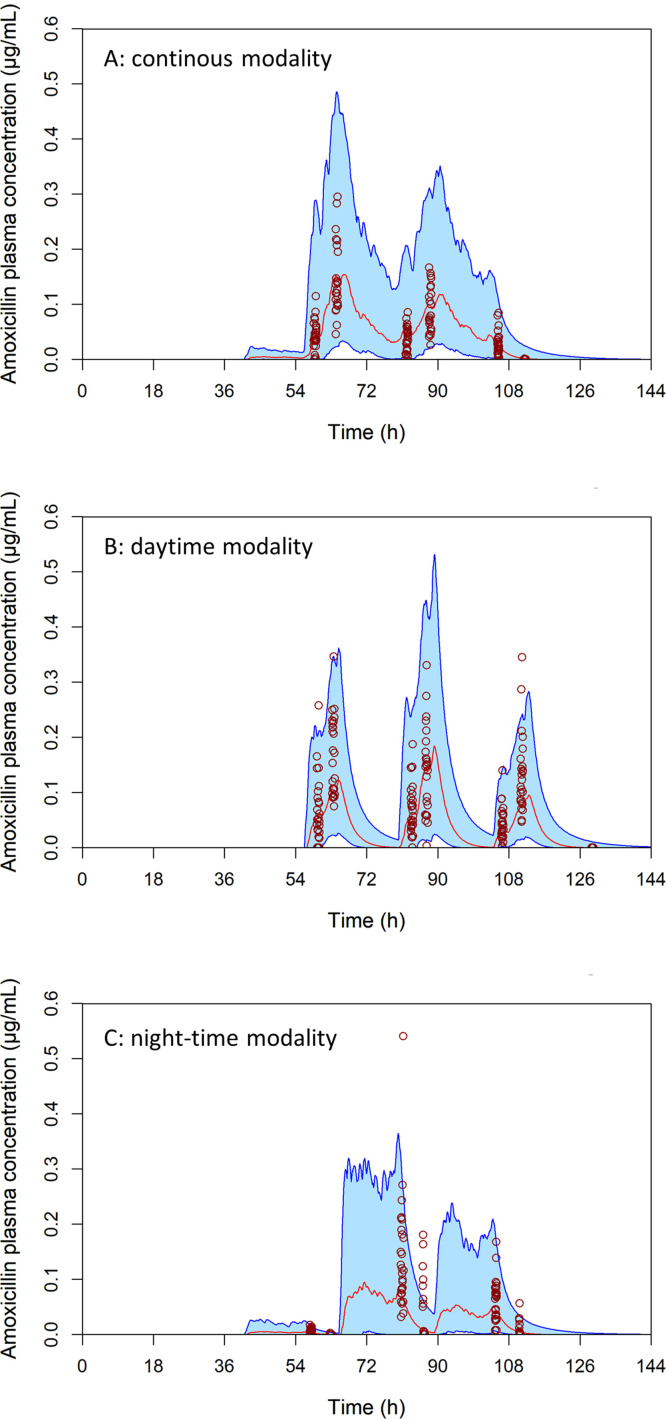
Modelling of plasma amoxicillin concentrations under the (A) continuous, (B) daytime, and (C) night-time modalities over 6 days [1.5 days before treatment, (A) 2.5 days or (B, C) 3 days of treatment, and (A) 2 days or (B, C) 1.5 days after]. Each simulation involved a theoretical amoxicillin treatment of 10 mg/kg/day administered either (A) continuously over 24 h, (B) during the day (8 am to 5 pm), or (C) during the night (5 pm to 8 am) for 3 days, to 5000 piglets per modality via drinking water. The lower blue curve represents the 5^th^ percentile and the upper blue curve the 95^th^ percentile; the light blue area between them illustrates the concentration range for 90% of the population. The red curve indicates the group median. Red circles show the individual plasma concentrations actually measured on the experimental farm in 51 piglets per modality.

Amoxicillin plasma concentrations for the 153 piglets sampled during the three treatment modalities on the experimental farm are shown in Fig. 4. In the same figure, these observed concentrations are compared with the model outputs based on piglets' actual water consumption, the measured concentrations in the drinking troughs, and the random draws of PK parameters. The observed concentrations were generally consistent with the simulated distribution, supporting the adequacy of the model for representing on‑farm exposure.

Visually, the experimental data points appeared to be well distributed on either side of the simulated median and across the range representing 90% of the simulations.

In addition to the visual verification of the predictions, we quantified the proportion of observed concentrations falling below the 5th percentile, within the range between the 5th and 95th percentiles, above the 95th percentile, and on either side of the median for each sampling time point and each treatment regimen (Table 4). Overall, between 31% and 48% of the observed plasma concentrations laid above the modelled medians (and therefore 69% to 52% below). For all treatment regimens, 85–100% of observations at sampling times in the middle of the profile felt within the prediction range, whilst deviations were mainly observed during the early absorption and late elimination phases.

**Table 4:** Proportion of observed concentrations relative to model predictions, by sampling time and treatment modality—continuous, daytime and night-time—with amoxicillin at 10 mg/kg/day.

Experiment stages	Sampling time	Modality	Below 5th (%)	Between 5th-95th (%)	Above 95^th^	Below-Above median (%)
2nd day	Am	Continuous	15 (4/27)	85 (23/27)	0 (0/27)	59-41 (16/27-11/27)
		Daytime	0 (0/25*)	96 (24/25*)	4 (1/25*)	60-40 (15/25*-10/25*)
		Night-time	0 (0/27)	100 (27/27)	0 (0/27)	48-52 (13/27-14/27)
	Pm	Continuous	0 (0/24)	100 (24/24)	0 (0/24)	71-29 (17/24-7/24)
		Daytime	0 (0/24)	100 (24/24)	0 (0/24)	33-67 (8/24-16/24)
		Night-time	0 (0/24)	100 (24/24)	0 (0/24)	96-4 (23/24-1/24)
3^rd^ day	Am	Continuous	4 (1/27)	96 (26/27)	0 (0/27)	70-30 (19/27-8/27)
		Daytime	4 (1/27)	96 (26/27)	0 (0/27)	48-52 (13/27-14/27)
		Night-time	0 (0/27)	93 (25/27)	7 (2/27)	19-81 (5/27-22/27)
	Pm	Continuous	0 (0/24)	100 (24/24)	0 (0/24)	58-42 (14/24-10/24)
		Daytime	4 (1/24)	96 (23/24)	0 (0/24)	42-58 (10/24-14/24)
		Night-time	50 (12/24)	25 (6/24)	25 (6/24)	67-33 (16/24-8/24)
4th day	Am	Continuous	0 (0/27)	100 (27/27)	0 (0/27)	56-44 (15/27-12/27
		Daytime	7 (2/27)	93 (25/27)	0 (0/27)	52-48 (14/27-13/27)
		Night-time	0 (0/27)	96 (26/27)	4 (1/27)	36-63 (10/27-17/27)
	Pm	Continuous	88 (21/24)	13 (3/24)	0 (0/24)	100-0 (24/24-0/24)
		Daytime	0 (0/24)	92 (22/24)	8 (2/24)	21-79 (5/24-19/24)
		Night-time	50 (12/24)	46 (11/24)	4 (1/24)	75-25 (18/24-6/24)
5th day	Am	Daytime	100 (27/27)	0 (0/27)	0 (0/27)	100-0 (27/27-0/27)

* Two samples were non‑quantifiable (NA)

Taken together, these results indicated that the model provided reasonable predictive performance for estimating population‑level exposure, particularly because low plasma concentrations contributed very little to AUC values, so the estimation of efficacy was not materially affected.

### 3.5 Simulations of 5-day amoxicillin treatment with the three treatment modalities

**Fig. 5:**
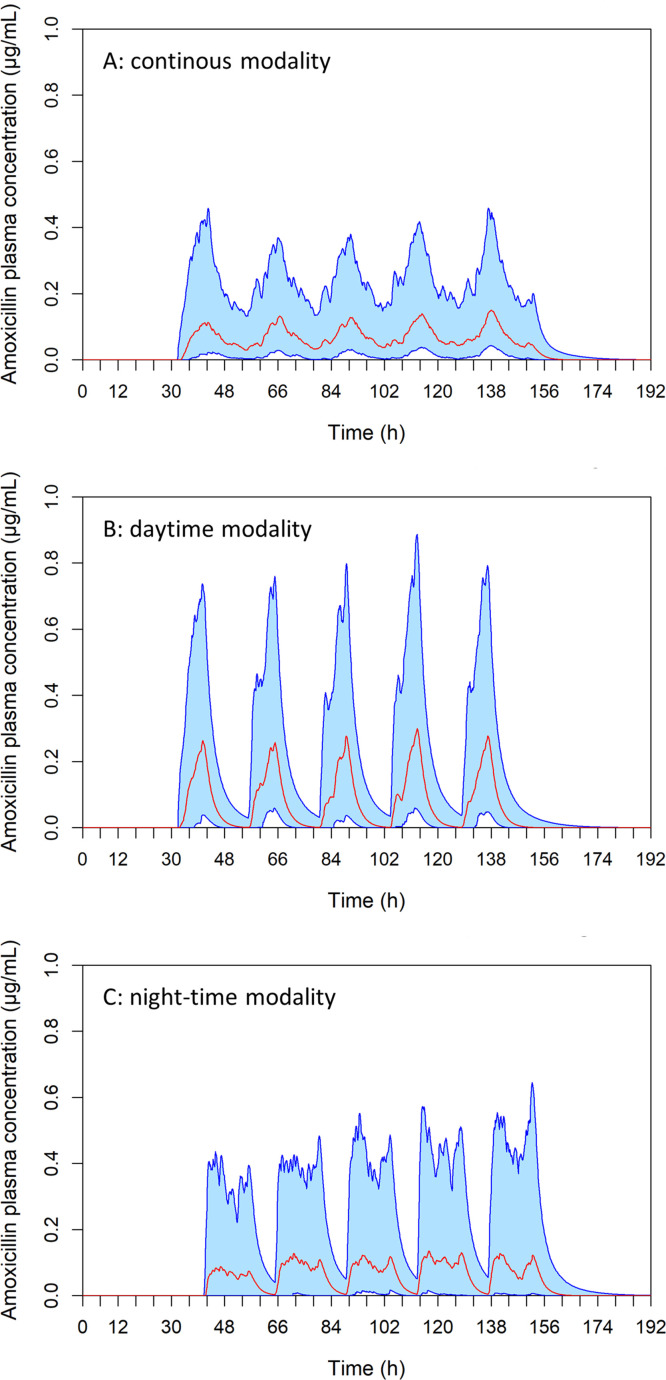
Modelling of plasma concentrations over 8 days (1.5 days before treatment, 5 days of treatment, and 1.5 days after), assuming that only 69% of the dose reached the drinking troughs. Each simulation involved an amoxicillin treatment (10 mg/kg BW/day) administered either (A) continuously (8 am to 8 am), (B) during daytime only (8 am to 5 pm), or (C) during night-time only (5 pm to 8 am) for 5 days, to 5000 piglets per modality. The lower blue curve represents the 5^th^ percentile and the upper blue curve the 95^th^ percentile; the light blue area between them shows the range of concentrations for 90% of the population. The red curve represents the group median.

The same model was used to simulate the three treatment modalities with amoxicillin delivered at 10 mg/kg BW/day for 5 days. Simulations assumed that only 69% of the amoxicillin reached the drinking troughs, as observed in the farm experiment. The results are presented in Fig. 5. The variation in plasma concentrations over time indicated that there was no accumulation of amoxicillin in plasma, irrespective of treatment modality.

**Fig. 6:**
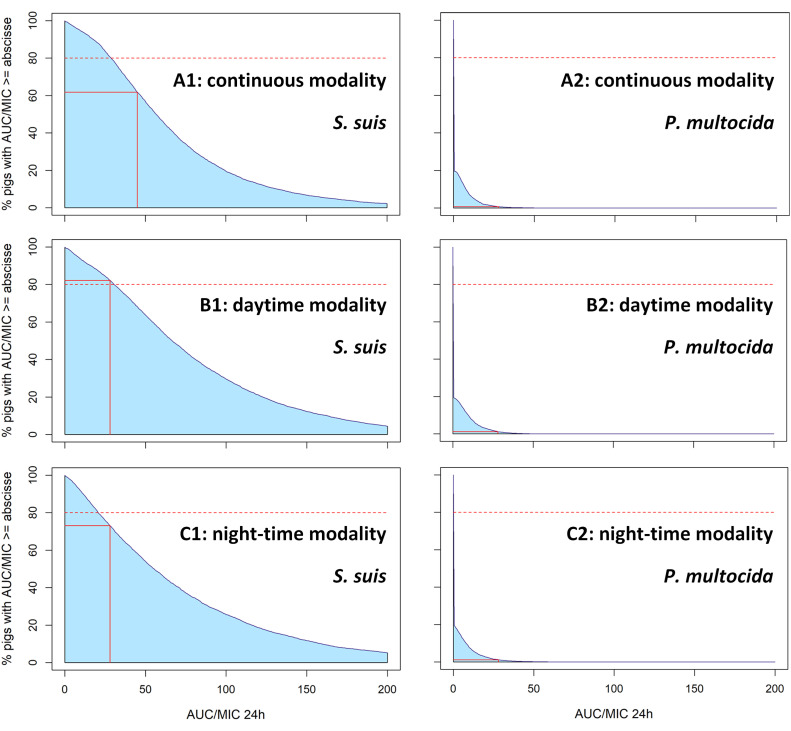
Percentage of piglets with AUC/MIC ≥ 28 h (bacteriostatic effect) for *Streptococcus suis* (left) and *Pasteurella multocida* (right) following amoxicillin treatment (10 mg/kg BW/day for 5 days) administered (A) continuously (8 am–8 am), (B) only during daytime (8 am–5 pm), or (C) only during night-time (5 pm–8 am). The blue curve represents the percentage of piglets achieving an AUC/MIC ≥ each x-axis value. The red solid lines indicate the AUC/MIC targets and the corresponding percentages of pigs treated. The red dotted line represents the group-le@#@vel target of at least 80% of animals reaching the individual PK/PD threshol.

AUC values for the last 24 hours of each treatment were used to estimate efficacy against the five respiratory pathogens of interest. An example of probabilities of target attainment (PTA) for *Streptococcus suis* (left panel) and *Pasteurella multocida* (right panel) for a bacteriostatic effect (AUC/MIC ≥ 28 h) under each modality (from top to bottom: A: continuous, B: daytime, C: night-time) is shown in Fig. 6. Only the continuous and daytime modalities exceeded the 80% target of pigs treated for *Streptococcus suis* (80.7% and 82.2% of pigs treated, respectively). For *Pasteurella multocida*, none of the three modalities exceeded 2% of pigs treated for a bacteriostatic effect. This example illustrates the marked differences in PTA between pathogens, with limited variation across dosing modalities for each pathogen.

Detailed results for each bacterium, modality, and effect are summarised in Table 5. As expected, all other factors being equal, the proportion of animals achieving a bactericidal effect was systematically lower than for a bacteriostatic effect. Furthermore, across all modality–effect combinations, the 80% threshold was met only for the continuous and daytime treatments for a bacteriostatic effect against *Streptococcus suis*. This target was not achieved for any of the four remaining pathogens (*Pasteurella multocida, Actinobacillus pleuropneumoniae, Bordetella bronchiseptica*, and *Glaesserella parasuis*), regardless of modality AUC/MIC target. Among these four, at least half of the animals achieved a bacteriostatic effect only for *Glaesserella parasuis*. For the other three pathogens, the proportion of treated animals remained below 4%.

**Table 5:** Percentage of piglets with AUC/MIC ≥ 28 h (bacteriostatic) or AUC/MIC ≥ 45 h (bactericidal) for all simulations under each modality—continuous (C), daytime (DT) and night-time (NT)—with amoxicillin at 10 mg/kg/day.

Effect		Bacteriostatic			Bactericidal		
AUC/MIC goal		28 h			45 h		
Treatment modality		C	DT	NT	C	DT	NT
Bacteria	*P. multocida*	0.7%	1.2%	1.2%	0.2%	0.2%	0.3%
	*A. pleuropneumoniae*	1.5%	3.0%	3.5%	0.2%	0.4%	1.1%
	*B. bronchiseptica*	0.0%	0.0%	0.0%	0.0%	0.0%	0.0%
	*G. parasuis*	52.6%	57.7%	50.6%	35.1%	41.4%	35.8%
	*S. suis*	80.7%	82.2%	73.1%	61.8%	68.1%	58.2%

## 4 Discussion

Among the antibiotics commonly used in pig farms, amoxicillin remains a key treatment for severe systemic infections such as *Streptococcus suis*, as well as major respiratory infections caused by *Actinobacillus pleuropneumoniae* and *Pasteurella multocida* (Burch & Sperling, 2018). Post-weaning is a particularly critical phase for piglets, with a high incidence of respiratory disease, and collective treatment via drinking water is common practice (Filippitzi et al., 2014; Timmerman et al., 2006; van Rennings et al., 2015). Despite the widespread use of amoxicillin for these pathogens via drinking water, several studies have shown that its efficacy can be compromised. For instance, Rey et al. (2014), using Monte Carlo simulations, demonstrated that even at the dose usually recommended for severe damage (i.e., 20 mg/kg/day, orally), the PTA of 90% was never achieved for the recommended breakthrough threshold of 0.5 µg/mL for *Actinobacillus pleuropneumoniae*. Even doubling the daily oral dose to 40 mg/kg/day only allowed a PTA above 90% at much lower MIC values. In this context, Hernández et al. (2024) suggested considering pigs’ watering habits to optimise the precise administration of treatment without increasing the total daily dose. A more refined optimisation of amoxicillin delivery—taking into account drinking behaviour—could increase the proportion of piglets effectively treated for *Pasteurella multocida* from around 30% to at least 60%, and from about 20% to more than 70% for *Actinobacillus pleuropneumoniae*.

However, such fine-tuned treatment management remains difficult to implement on pig farms because drinking behaviour cannot yet be predicted hour by hour. In practice, an hourly approach is even more challenging because daily water-intake patterns shift over time and can differ between pens within the same batch (Little et al., 2023), raising questions about the relevance of very narrow dosing windows if the aim is to develop a strategy applicable at farm level. In addition, concentrating the daily dose into too short a time window would also raise solubility issues for amoxicillin. For example, if the 3 pm–5 pm period in our farm experiment were targeted—representing only 15% of the daily water intake—while maintaining a daily dose of 10 mg/kg and assuming an incorporation percentage of 20% from the pump into the water line (to avoid overly concentrating the pump solution), the pump concentration would reach 3.3 mg/mL. This far exceeds the solubility limit of amoxicillin trihydrate (the form used for administration via drinking water) which is 2.6 mg/mL at 19°C and pH 7.7 (de Moraes et al., 2022).

We therefore developed a pharmaco-statistical model that accounts for individual drinking behaviour but follows an approach more readily applicable under field conditions. For the first stage of model building (determining PK parameters of amoxicillin in piglets), we chose a simple PK model. This choice was supported by Chassan et al. (2021), who showed that variability in PK parameters has only a limited influence on plasma exposure following antibiotic administration via drinking water. Our choice was further supported by the agreement between our PK parameter estimates and values reported in the literature.

The clearance estimated in our study (0.79 ± 0.07 L/h·kg) was of the same order of magnitude as the clearance of 0.49 ± 0.19 L/h·kg reported by the Anses report (Bousquet-Melou et al., 2017). The amoxicillin oral bioavailability was meanwhile estimated at 25% with a CV of 47%. Since the galenic formulation and the clinical health status were similar for all piglets in our study, these factors, which are known to significantly influence bioavailability in pigs (Anfossi et al., 2002; Dai et al., 2017; Hernández et al., 2005), cannot explain the observed dispersion. Instead, this high variability is consistent with the general pharmacological principle that inter-individual variability tends to increase as the extent of drug absorption decreases (Hellriegel et al., 1996). This is also in line with the meta-analysis of published data by Anses (Bousquet-Melou et al., 2017), which reported an average F of 35% with a CV of 41% in pigs.

In pigs, the influence of the prandial state on amoxicillin absorption remains also a subject of discussion regarding its effect on the mean bioavailability; while some authors found no statistically significant reduction in amoxicillin oral bioavailability (Agersø & Friis, 1998a), others reported a significant decrease of 37% to 45% in fed piglets (Decundo et al., 2024; del Castillo et al., 1998). Moreover, there is a consensus that the presence of food increases the variability of the absorption process and modifies the kinetic profile (lower C_max_ and delayed T_max_) (Agersø & Friis, 1998a; del Castillo et al., 1998). Since pigs in our study had ad libitum access to feed, they were in heterogeneous prandial states at the time of drug ingestion. This heterogeneity likely acted as a major source of the observed variability in F, reflecting real-world farming conditions rather than a methodological artefact. This high variability is therefore necessarily reflected in the simulated exposure profiles, which match pretty much actual experimental farm plasma concentrations, allowing it to be taken into account in the PTA.,

In a second step, modelling of amoxicillin plasma concentrations during our farm experiment was carried out using the PK parameters obtained under laboratory conditions, together with the amoxicillin concentrations measured in the drinking troughs and the individual real-time water consumptions recorded on the farm. Comparison of simulated data with observed plasma concentrations confirmed that our model was relatively well suited to representing the plasma concentrations obtained in the field: the measured concentrations were generally well distributed on either side of the modelled median and fell within the variability range for 90% of simulated individuals. Only the initial absorption and elimination phases appeared slightly overestimated. Indeed, between 0% and 7% of observed plasma concentrations exceeded the 95^th^ percentile, and between 16% and 17% fell below the 5^th^ percentile. This may be explained by actual concentrations during these phases being below the lower LOQ and therefore set to zero by default in the model. However, such potential bias had a negligible impact on overall exposure as described by the AUC and therefore did not materially distort estimates of therapeutic efficacy.

Once selected, the model was used to simulate continuous treatment with 10 mg/kg/day of amoxicillin over 5 days, corresponding to the traditional dosing regimen for respiratory diseases in post-weaning piglets. We showed that predicted PK/PD target attainment with this regimen was largely suboptimal for *Pasteurella multocida, Actinobacillus pleuropneumoniae*, and *Bordetella bronchiseptica*. Whether aiming for a bacteriostatic or a bactericidal effect, fewer than 2% of piglets were adequately exposed. This is lower, but still consistent with the findings of Hernández et al. (2024), who obtained only 20% and 30% of piglets meeting a target of AUC/MIC ≥ 25 h for *Actinobacillus pleuropneumoniae* and *Pasteurella multocida*, respectively, after administration of 20 mg/kg/day via drinking water. For *Glaesserella parasuis*, amoxicillin PTA was somewhat better, with 52.6% and 35.1% of piglets reaching bacteriostatic or bactericidal targets, respectively. Finally, continuous amoxicillin treatment at 10 mg/kg/day over 5 days reached predicted PK/PD thresholds at meaningful levels only for *Streptococcus suis*, as 80.7% (bacteriostatic) and 61.8% (bactericidal) of piglets achieved the PK/PD target.

We then assessed whether accounting for collective water-drinking behaviour could improve exposure while maintaining treatment modalities that remain feasible on farms. Several studies have reported higher water consumption during daylight, with peaks in late morning and afternoon (Larsen & Pedersen, 2022; Little et al., 2023; Massabie et al., 2014; Ramonet et al., 2019). This was also observed in our farm experiment, with higher water intake between 11 am and 7 pm. We therefore simulated a daytime-concentrated treatment, delivering the same daily dose (10 mg/kg/day) only between 8 am and 5 pm (i.e., 10 mg/kg/9 h). Conversely, we simulated a night-time treatment (10 mg/kg/day delivered from 5 pm to 8 am; i.e., 10 mg/kg/15 h). Despite low and irregular water consumption at night—reflected in both the literature and our data (Andersen et al., 2014)—this modality was considered worth testing under the hypothesis that individual consumption might be more regular during periods of reduced collective activity (Rousselière et al., 2016).

Overall, these two alternative modalities did not meaningfully improve predicted exposure or PK/PD target attainment. For night-time treatment, the percentage of animals achieving a bacteriostatic effect against *Glaesserella parasuis* or *Streptococcus suis* even decreased slightly (from 52.6% to 50.6% for *Glaesserella parasuis* and from 80.7% to 73.1% for *Streptococcus suis* when comparing continuous with night-time modality). This was unsurprising given the persistently low water consumption at night; combined with the short half-life of amoxicillin, this made it impossible to reach high plasma concentrations. Daytime treatment showed a modest improvement compared with continuous one, with an additional 5.1% and 6.3% of piglets reaching the bacteriostatic or bactericidal PK/PD target, respectively. However, overall predicted plasma exposure remained insufficient, and—as with continuous treatment—the 80% threshold was reached only for a bacteriostatic effect against *Streptococcus suis*. Thus, concentrating the dose during daytime, while more practical on farms than the ‘daily-fractionated’ strategy proposed by Hernández et al. (2024), does not align closely enough with actual consumption peaks.

This study was conducted in healthy, recently weaned piglets of similar age and body weight, which differs from the heterogeneous physiological and health status typically encountered during field outbreaks. However, disease can influence both water‑drinking behaviour and the pharmacokinetics of orally administered drugs (Little et al., 2019). Although water intake is generally less affected than feed intake during infectious episodes (Gagnon et al., 2018; Little et al., 2019; Thomas et al., 2021), some respiratory diseases can actually reduce drinking behaviour (Pijpers et al., 1991). In addition, while water intake remains broadly proportional to body weight (approximately 10% of BW), circadian drinking patterns become more pronounced as pigs grow (Little et al., 2023; Thomas et al., 2021). Importantly, weight heterogeneity in commercial herds also often amplifies social competition at drinkers, with social rank sometimes being a stronger determinant of individual exposure than body weight itself (Little et al., 2019; Toutain et al., 2021). Under field conditions, dosing calculations should therefore ideally rely on water‑consumption data recorded immediately before treatment. By adjusting the drug concentration to the actual volume of water consumed, the administered dose (mg/kg) would remain stable, and the influence of physiological variability is then expressed only through pharmacokinetic parameters. Regarding pharmacokinetics, respiratory infection has been shown to markedly increase the oral bioavailability of amoxicillin in pigs—up to a three‑fold rise in some cases (Godoy et al., 2010). Using healthy animals in our study may therefore underestimate systemic exposure in diseased pigs, although during metaphylaxis, most animals have not yet reached advanced clinical stages. Furthermore, the use of piglets with homogeneous age and body weight represents a simplification of field conditions. Body weight is a key covariate for PK parameters: clearance and volume of distribution increase with body mass, which tends to prolong the elimination half‑life of antibiotics in heavier animals (Toutain et al., 2025). Older pigs could therefore exhibit longer plasma persistence than predicted by our model based on young piglets.

Another pharmacokinetic aspect to consider is plasma protein binding (PPB). Only the unbound fraction of an antimicrobial is microbiologically active, as it can distribute into interstitial fluids where most bacterial infections occur. However, amoxicillin exhibits a low PPB in pigs, with unbound fractions ranging from 76% to 83% in healthy or *Actinobacillus pleuropneumoniae*‑infected animals (Agersø & Friis, 1998b; Agersø et al., 1998). According to Toutain et al. (2002), correction for protein binding is mainly required when the unbound fraction is below 20%, which is not the case for amoxicillin in this species. Recent work further indicates that, for antimicrobials with low to moderate PPB, total plasma concentrations adequately predict interstitial fluid exposure (Toutain et al., 2021; Bidgood & Papich, 2005). In our study, most pathogen–antibiotic combinations did not reach PK/PD targets even when using total concentrations, and using free concentrations would therefore not alter the conclusions. The only borderline case could be *Streptococcus suis*, for which the bacteriostatic target was reached in slightly more than 80% of piglets; however, given the low PPB of amoxicillin and the availability of a higher recommended dose (20 mg/kg/day), sufficient exposure can still reasonably be achieved in practice.

Our model should thus be viewed as providing a conservative baseline (a worst‑case scenario for respiratory disease), while acknowledging that real‑world PK/PD performance may vary depending on the physiological and health status of the herd.

Nevertheless, it would be of interest to use our model to evaluate different strategies, like the daytime one, with other antibiotics commonly used for collective treatment of porcine respiratory disease, such as doxycycline, which has higher oral bioavailability and slower elimination than amoxicillin (Bousquet-Melou et al., 2017).

## 5 Conclusion

Our study relied on a controlled protocol in healthy pigs of uniform age, enabling the development of a pharmaco-statistical model consistent with field observations. Its application to amoxicillin administration via drinking water in post-weaning piglets revealed significant PK/PD limitations in the current dosing regimen, and testing alternative modalities such as daytime or night-time administration did not improve predicted PK/PD efficacy at the group level.

Nevertheless, this pharmaco-statistical model is a powerful tool that could be adapted to other antibiotics and other animal species, such as poultry or small ruminants, provided species-specific water-consumption profiles, PK parameters, and MIC distributions of the targeted pathogens are available. Its predictive value could also be strengthened by incorporating covariates influencing water consumption or PK, such as age, social hierarchy, digestive tract filling, or health status.

## Glossary

AUC/MIC: PK/PD index relating drug exposure over 24 h to bacterial susceptibility.

BLQ: Below the limit of quantification; concentration too low to be reliably measured.

ESI+: Electrospray ionisation in positive mode (mass spectrometry).

LC‑MS/MS: Liquid chromatography coupled with tandem mass spectrometry.

Lognormal distribution: Distribution where the logarithm of the variable follows a normal distribution.

Logit‑normal distribution: Distribution used for parameters bounded between 0 and 1 (e.g., bioavailability).

MRM: Multiple reaction monitoring, a selective MS acquisition mode.

PTA (Probability of Target Attainment): Probability that a dosing regimen reaches the PK/PD target in a population.

## Ethical approval

The protocols were authorised by the French Ministry of Research under the number #44369_202308070921823 for the laboratory experiment carried out at the INTHERES animal facility, and #46144_2023112916569025 for the experiment performed at the IFIP experimental farm.

## Data availability statement

The data that support the findings of this study are available from the corresponding author upon reasonable request.

## Funding sources

This study was funded by the PigPills project under the Carnot France Futur Élevage framework.

## Disclosure statement

The authors declare no competing interests.

## Ethical statement

The protocols were authorised by the French Ministry of Research under the number #44369_202308070921823 for the laboratory experiment carried out at the INTHERES animal facility, and #46144_2023112916569025 for the experiment performed at the IFIP experimental farm.

## CRediT authorship contribution statement

**Marine Lacampagne:** Writing – review & editing, Writing – original draft, Methodology, Investigation, Conceptualization. **Gwendoline Hervé:** Writing – review & editing, Supervision, Methodology, Conceptualization. **Lucie Claustre:** Writing – review & editing, Formal analysis. **Natasha Jourdannaud:** Writing – review & editing, Investigation, Formal analysis. **Marlène Lacroix Z.:** Writing – review & editing, Writing – original draft, Project administration, Methodology, Investigation, Formal analysis, Conceptualization. **Béatrice Roques B.:** Writing – review & editing, Writing – original draft, Supervision, Project administration, Methodology, Investigation, Conceptualization.

## Declaration of competing interest

The authors declare no competing interests.
